# Identifying Factors Influencing Productivity of Older Workers in Service Sector: A Case Study in Pilot Companies in Thailand

**DOI:** 10.3390/bs12080268

**Published:** 2022-08-04

**Authors:** Chonticha Asavanirandorn, Watchara Pechdin, Nguyen Thi Quynh Trang

**Affiliations:** 1 College of Population Studies, Chulalongkorn University, Bangkok 10330, Thailand; 2 Disaster Preparedness, Mitigation, and Management Program, Asian Institute of Technology, Pathum Thani 10120, Thailand; 3 AIT Center for Global Challenges, Asian Institute of Technology, Pathum Thani 10120, Thailand

**Keywords:** older worker, productivity, service sector, gologit, employment, promoting work, development, Thailand

## Abstract

Productivity has posed issues for global countries in terms of promoting older employment due to an emergence of questions regarding production efficiency. Individual characteristics result in varying production efficiencies, which in turn provide different levels of productivity. Taking this concern into account, we are here to examine the characteristics of older workers who provide high productivity in order to seek recommendations for fostering decent work for them. We utilized the dataset collected from the pilot service companies in Thailand who participated in a national initiative program for employing older people. A total of 204 older workers and their characters were then observed. Those characters were analyzed by the Generalized Ordered Logit Regression (gologit) model. Empirical findings indicate that allocating work that is related to communication and coordination to older workers would enhance odd ratio of their productivity by as much as 4.79 times compared to general tasks. Furthermore, employing older individuals on a part-time basis tends to generate higher-level productivity than full-time employment. We also found that gender and age differences have no significant effect on productivity in the service sector as people age, and factors related to types of employment, education, health, and financial status are also a precise determinant for the productivity of older workers. This would suggest that the work design for the older workers must be in line with the aforementioned determinants. In addition, the government initiatives utilizing key findings from this study to boost older workers’ productivity should give priority on tax incentives, promoting and advocating for employment equality, and vocationalization programs.

## 1. Introduction

Since an older population imposes a cost on society, especially in terms of economic activity, concern has risen sharply for a country’s economic development as its society ages [1,2,3,4]. Thus, several countries have begun enacting aging policies aimed at prolonging the working lives of their workforce, including extending the retirement age, introducing incentives for employers to employ aging labor, or providing upskilled or reskilled training to those who have already retired in order to keep them working or to return them to the labor market [5,6,7,8].

Thailand facing labor shortages influenced by a growing aging society in the early 2000s has put in place return-to-work policies to promote the employment of older workers after they hit 55 years old, which is the first year of retirement age in a private sector [9]. Although on the one hand, this is an attempt to mitigate labor shortages in domestic production, on the other hand, some of them are not yet prepared for retirement owing to a lack of stability in their lives, primarily due to them being stuck in a “poverty trap”. The opportunity to continue working would help them have income, thereby becoming less of a burden on their children or family.

However, employability is fundamentally influenced by worker productivity, as represented by the degree of efficiency of production of goods or services. Thus, if older workers perform less productively, their employability would indeed be limited. Therefore, it is essential to understand what factors determine their productivity.

Even though some studies have a discussion on factors influencing labor productivity [10,11,12,13,14], there is room for specific study inquiring into this issue in the context of older workers. Our study here thus addresses this gap through a case study that introduces evidence from the employment of older workers in Thailand. We put our case study on the service sector as older workers tend to prefer this work because the nature of the work is service-oriented [15,16] and does not directly degrade their physical health. Therefore, promoting the service sector would be possible to use as a guideline for creating work opportunities for the older workers who choose to continue working. 

This produced contributions from our study to emphasize the factors influencing older workers’ productivity, which is important for the first step of policymakers or relevant stakeholders to support decent employment for older people. The rest of the study is divided into five sections. Firstly, we provide an overview study regarding the definition of productivity and its determinants. After that, the methodology, including data collection, variables, and the econometric model to investigate the characteristics of older workers’ productivity, are presented followed by the empirical findings. Then, the study discussion is addressed, followed by suggestions for how to proceed going forward and how Thailand, including other countries, can promote and enhance productivity in older workers. 

## 2. Relevant Studies: Defining Productivity and Its Determinants 

In this section, we aim at delivering an overview of the productivity concept and its determinants. Productivity is typically described as the ratio of output to input for producing one unit of product or service. It is used as a measurement for determining the efficiency of a production process. Its calculation varies depending on how the outputs and inputs are defined [17,18]. Tolentino [19] indicated that productivity should be measured differently, mainly due to the nature of the output, as some measurements are principally dependent on consumer preference and utility, which has an unclear countable output [20]. Concisely, productivity should be narrowed down as the ratio of products and services produced, and the efficiency with which they are produced and delivered to customers. 

This is just the case for the overview of the productivity measurement. When considering measuring the productivity of labor, defined as the ratio of quantitative output to one unit of labor, it has been recognized as a critical concern for employment. Which factors have more of an impact on labor productivity has been a topic widely discussed, and it has been acknowledged that this productivity is related to age, gender, education, skill set, and working conditions, as well as personal needs [1,21,22,23,24,25,26,27,28,29,30,31]. In terms of age, it has been generally observed that this factor potentially influences the recruitment of a workforce due to an assumption of a significant change in their productivity [1,21,23,24,28]. In contrast, this assumption has been challenged as not being the case in the service sector. According to Göbel and Zwick [22], who examined the age and productivity in manufacturing, metal manufacturing, and the service sectors with the estimation of a generalized method of moments (GMM), key findings stress that there was no significant variation in productivity in service sectors across age groups. In contrast, younger age groups could likely exhibit lower productivity relative to older workers. Supported by Börsch-Supan, Hunkler [32] found that there was no evidence of productivity reduction in ages between 20 and 60, and that the productivities even improved at the age of 60–65. The recommended productivity in the service sector was not found to be affected by age alone. 

As for the gender factor, several scholars have also provided evidence of differences based on gender in measuring productivity in the service sector [22,26,33]. For example, Göbel and Zwick [22], found only an insignificant effect arising from gender differences among workers in the service sector. However, in other industries, manufacturing in particular, significant effects on productivity based on gender differences were discovered [33]. This was also confirmed by Pfeifer and Wagner [25], who studied gender and age in manufacturing industries in Germany and stated that there was a significant effect on productivity in manufacturing based on gender—with females exhibiting lower productivity than males. 

Looking into the role and impact of education on productivity, some evidence confirms that differences in educational levels could lead to a variation in productivity in the service sector [30,34,35,36]. For example, according to Van Dalen, Henkens [30], there is no significant effect of middle education level (aged between 10–15 years old) on productivity between younger and older workers. However, at higher educational level, older workers were significantly more likely to boost productivity of a firm rather than younger workers. In addition to the factor of working conditions, there is no exact inclination for whether part-time or full-time working is suitable for older workers [22,37,38,39]. Göbel and Zwick [22] showed that working part-time would provide positively significant effects on the productivity of workers in the service sector, lower effects in manufacturing industries, and no significant effect on the metalworking sector. Unlikely, Børing and Grøgaard [37] and Bryson, Forth [39] found negative impacts from working on a part-time basis on older productivity as there are rising concerns over work continuity. 

Meanwhile, investigations into the relationship between skills and productivity among older workers have found no specific significant relationship. Turek and Perek-Bialas [29], who compared the productivity between younger and older employees across industries, found that younger employees were more productive in the service sector than older workers in terms of hard skills such as computer skills, data analysis, or specific knowledge, whereas the older workers tended to be more productive in terms of soft skills such as communication, coordination, or interpersonal skills. However, within the older and their cohort, Göbel and Zwick [22] reported that productivity in the service sector significantly decreased if those employees were unskilled. 

Another considerable factor influencing productivity among the older workers is their motivation to return to the labor market, specifically as it relates to their health and financial status. In terms of health, older persons who are motivated to maintain a healthy lifestyle probably contribute more work productivity since their job functions may offer them an opportunity to stay active, thereby improving their health status [27]. As for financial status, some researchers found that there are significant impacts on older workers’ productivity. A study by Kaur, Mullainathan [31], for example, indicates that workers would be less productive precisely when they are most in need of money, paying less attention to the work. 

In conclusion, a review of the literature suggests that productivity might be determined to a certain degree by workers’ demographic characteristics, working conditions, or other related factors. Thus, while conducting research regarding labor productivity, these aforementioned factors should be considered.

## 3. Method

### 3.1. Dataset and Limitation

As most of the workplace has not been widely adopted for the employment of older workers, especially in the service sector, this circumstance has challenged us to reach older workers for collecting this information. This paper, then, utilized the dataset with permission from the research project of “*Non-Standard Employment for Older Persons in the Thai Private Sector: Flexibility Productivity and Protection*” by Asavanirandorn, Osatis [40]. The project was funded by the National Research Council of Thailand (NRCT) and the Foundation of Thai Gerontology Research and Development Institute (TGRI). The project collected data via a questionnaire survey, which is in accordance with the Declaration of Helsinki, and was approved by the relevant ethical committee, conducted in 2020 from older workers in service companies that signed a Memorandum of Understanding (MOU) with the Thai Government under the project “Pracharat (E6)”. The Pracharat (E6) project was implemented in 2017 to provide decent work opportunities for older people in Thai society. Under this initiative, participating companies were asked to rearrange working environments and adjust job responsibilities to accommodate for older workers. These conditions could ensure that factors impacting older productivity would be free of physical environment concerns and mismatching skills at work.

Referring to that project, they use relative employer’s perception of older workers’ productivity to predict the productivity of individual older workers. Since an employer is strongly engaged with an employee, they hypothesized this relationship would provide reasonable estimation. This was done to solve the difficulty in measuring output in the service sector, where it is influenced by customer preference, utility, happiness, convenience, or comfortable feelings. The data were collected through a questionnaire completed by employers or supervisors. Each employer or supervisor was asked to figure out information concerning both older workers and general (younger) workers performing the same jobs in order to compare levels of productivity within similar work situations. 

In total, 204 older persons were observed by their employers/supervisors. These were out of a total of 972 older persons who registered as older workers in the MOU companies. The occupations of observed older workers highlighted service-intensive tasks such as customer services, sales representative, merchandiser, or supervisor. The estimated sampling error determined from the Yamane method was reported to be approximately 5%. 

This dataset has formed limitations for our study. A total of 204 observations are challenging to us for describing the analysis. However, we attempt to minimize this sample-size effect by constructing a hypothesis that tests for data distribution and by choosing to appropriate the technique for them. Extensive literature reviews were included into the discussion for further consideration on key findings. This is done to ensure that our analysis is sufficient to explain and answer the objectives of the study. 

### 3.2. Variables

#### 3.2.1. Productivity

According to the dataset, their predicted productivity was calculated as a percentage that ranged from −100% to +100%, with a minus sign denoting that the employer considered a particular older worker to have lesser productivity than a younger worker in the same task and occupation, whereas a plus sign denoted the opposite.

Since the focus of this study is to identify the characteristics of older workers who meet a high level of productivity, the productivity as a dependent variable should perform better in the form of a categorical variable to indicate the degree of high productivity. Consequently, we have to categorize it. We normalized those numbers by generating distributed proportions of three levels of perceptions on productivity, namely, dissatisfied (−100 to −35), indifferent (−34 to +35), and satisfied levels (+36 to +100). The dissatisfied level is cited for an individual older worker who has productivity that is lesser than a younger worker in the same task and occupation, whereas the indifferent level means there is no significant difference between the younger and older productivity in the same task and occupation, and the satisfied level is defined as the older individual having higher productivity as compared to the younger in the same task and occupation. 

#### 3.2.2. Factors Determining Productivity

Upon considering the correlations between the productivity and its determinants, we selected seven variables to be analyzed including gender, educational level, working basis, occupational characteristics, self-perceived health status, and financial status, as described in Table 1 as follows:

### 3.3. Empirical Model

Beginning with the considerations on the specific characteristics of the dependent variable, namely, the productivity of older workers, the Ordered Logit Regression model (ologit) was firstly considered. The model is fundamentally used for analyzing the relationship among ordered categories, which might be in the form of “poor”, “fair”, “good”, “very good”, or “excellent”. Therefore, its calculation, which is an extension of the Logit Regression which is used only for a dichotomous variable, is to estimate by the probability of being one category compared to all the lower or higher categories.

Although the ologit is useful for analyzing ordered categorical data, disregarding its key assumption may affect its conclusion. The main assumption of the ologit model is the proportionality assumption, which is that the distance between each outcome category is proportionate. The proportionality assumption of the ologit model can be examined in various ways. One famous technique was proposed by Brant [41]. The Brant test is described as a combination of k−1 logit regression where k is denoted as the total number of categories of dependent variables.

(1) $$y_{j}=\{\begin{array}{cc} 1 & y>k\\ 0 & y\leq k \end{array}$$

Brant demonstrated that when the null hypothesis is accepted, the independent maximum likelihood of estimating its coefficients has an asymptotically multivariate normal distribution [42]. Thus, the Wald test statistic, which is based on the correlated separate fits and distribution chi-square $\left(\chi^{2}\right)$, could provide an assessment of the proportional assumption. His $\chi^{2}$ is obtained by Equation (2):

(2) $$\chi^{2}={\left(D\tilde{\beta }\right)}^{t}{\left[D\hat{V}\left(\tilde{\beta }\right)D^{t}\right]}^{-1}\left(D\tilde{\beta }\right)$$

where $\hat{V}\left(\tilde{\beta }\right)$ is a covariance matrix of proportional odds ($COV\left({\tilde{\beta }}_{i}{\tilde{\beta }}_{j}\right)$), which $\tilde{\beta }$ is a coefficient matrix estimated by the maximum likelihood and i≠j. In terms of D, it is a matrix denoted as Equation (3):

(3) $$D=\left[\begin{array}{cccc} I & -I & 0 & \ldots \\ I & 0 & -I & \ldots \\ \ldots & \ldots & \ldots & \ldots \\ I & 0 & 0 & \ldots \end{array}\begin{array}{c} 0\\ 0\\ \ldots \\ -I \end{array}\right]$$

If the calculated $\chi^{2}$ is found to be significant, it means that the individual differences ${\tilde{\beta }}_{i}-{\tilde{\beta }}_{j}$ are probably considered in relation to their approximate standard errors to elucidate the nature of the lack of fit [41]. This means that it rejects the proportional odds assumption hypothesis (null hypothesis), indicating that there is a bias of proportionality in a dependent variable and the proportionality assumption is not satisfied. Therefore, the ologit model might not provide a good estimation. 

### 3.4. Generalized Ordered Logit (gologit) 

In any case where there is a violation on the proportionality assumption, the model can be estimated in another perspective by the Generalized Ordered Logit (gologit) developed by Fu [43]. 

In productivity analysis with emphasis on economic and behavioral aspects, the gologit model has been widely adopted, e.g., [44,45,46]. The model is an extension of the ologit by relaxing the proportional assumption. It can be written corresponding to a gologit assumption in the general form as Equation (4):

(4) $$P(Y_{i}>j)=\frac{e^{\beta_{j}X_{i}}}{1+e^{\beta_{j}X_{i}}},j=1,2,\ldots ,m-1$$

where *Y*, *X* are the dependent and independent variables, respectively. m is the total number of categories of dependent variables. It can be developed to our analysis as Equations (5) and (6):

(5) $$P(AP>j)=\frac{e^{\beta_{j}X_{i}}}{1+e^{\beta_{j}X_{i}}},j=1,2$$

where

(6) $$X_{i}=\alpha_{0}+\beta_{1}AGE_{i}+\beta_{2}GDR_{i}+\beta_{3}EMP_{i}+\beta_{4}SKS_{i}+\beta_{5}EDU_{i}+\beta_{6}HSS_{i}+\beta_{7}FNS_{i}$$

$AP_{i}$ is the individual productivity of the older person, and i, j is the level of satisfaction with an older worker’s productivity. Since AP is denoted as three categories, the gologit model for this study will end up having two sets of coefficients (m = 3), we hence scored as dissatisfied (j=1), indifferent (j=2), and satisfied (j=3). The dissatisfied level refers to an older worker’s productivity not satisfying their employer’s expectation when compared to the productivity of the general labor pool in the same occupation. The indifferent level refers to the productivity of the older worker that is not significantly different from the productivity of the general labor pool in the same occupation. Lastly, the satisfied level refers to the older worker whose productivity satisfies employer expectations when compared to workers in the same occupation.

Meanwhile, X is defined as a set of productivity determinants that consists of the age of the aging person (AGE), gender (GDR), type of employment (EMP),occupation set (SKS), education level (EDU), health factor (HSS), financial status (FNS), and β,α is a set of coefficients.

In terms of its interpretation, Equation (5) is interpreted as the *odd ratio*, indicated in Equation (7) as follows:

(7) $$\frac{P\left(AP_{i}\geq k+1\right)}{P\left(AP_{i}\leq k\right)}=\chi_{0}+\chi_{i}X_{i}+\varepsilon_{i}$$

where k=1, 2 and χ is a set of coefficients of the odd ratio, and ε is disturbance terms. 

According to Equation (7), the probability of an employer being satisfied with the level of productivity of an individual older worker i is determined by χ in the set X. For example of interpretation, considering a discrete factor such as $\chi_{1}AGE$ in the set X of Equation (4), if the age of the older worker i increases by one year, the odd ratio of probability of the employer reaching AP=3 versus AP=2 and AP=1 is $\chi_{1}$, holding other variables constant. Simultaneously, if the age of the older worker *i* increases by one year, the odd ratio of probability of the employer reaching AP=3 and AP=2 versus AP=1 is $\chi_{1}$, holding other variables constant. In term of a dichotomous variable, as well as categorical variables, its coefficient can be interpreted by comparing each category over a based category (comparing with 1). For example, considering $\chi_{2}Gen$ by giving female as a based category, when $\chi_{2}$ is less than 1, which means that the odd value contributed by a male is less than that of a female, it could indicate that changing from male to female reduces productivity by (1 − $\chi_{2}$). Unit of the coefficients of each predictor in the set X can be described in the form of “percentage” when it multiplies with 100.

## 4. Results

### 4.1. Descriptive Statistics

Table 2 summarizes the data obtained from Asavanirandorn et al. (2020), including gender, age, education, type of employment, health, financial status, and productivity of older workers in detail. Data were collected from 204 individuals: 76 males (37.3%) and 128 females (62.7%). The age distribution was from 55 to over 84 years, although the largest group was from the 60–64 age group (56.4%). The education level of the sample varied, from primary school to bachelor’s degree or higher. Of the sample, 148 people (72.5%) had less than an undergraduate education and 56 people (27.5%) held a university degree or higher. 

Older workers were involved in various kinds of employment, with the majority working full-time (113, or 55.4%) followed by those working part-time (91, or 44.6%). Regarding occupational characteristics, general task workers comprised the largest portion of older workers (108, or 52.9%), followed by intensive communication/coordination workers (56, or 27.5%) and specific task workers (40, or 19.6%). In terms of perceived health status, 161 people (78.9%) reported they were in good condition while 43 people (21.1%) reported uncertain or poor condition. In addition, 117 older workers reported that they were satisfied with their financial status, compared with 87 who reported being dissatisfied.

In terms of productivity, employers/supervisors were “satisfied” with 50% of their older male workers (38 individuals) as well as with 50% of their older female workers (64 individuals). When age is taken into account, 57.1% of older workers whose age is lower than 60 could reach the satisfied level, whereas workers whose ages are 60–64 years old and older workers over the age of 64 could reach this level only 53.0% and 41%, respectively. With regard to education, employers/supervisors were satisfied with the 70 people (47.3%) who lacked an undergraduate education and were satisfied with the 57.1% of those with an undergraduate or higher degree. Of the 113 older workers working full-time, only 70 (47.3%) were deemed “satisfactory” by their employers/supervisors in terms of their productivity. 

A slightly greater percentage of part-time older workers were similarly deemed satisfactory (44 out of 91, or 48.4%). There are also significant differences in terms of occupational characteristics. Intensive communication/coordination and intensive specific knowledge older workers, which comprised the majority of workers, were able to achieve a “satisfied” level by 33.3% and 39.4%, respectively. In contrast, the majority of general task workers reached the “dissatisfied” level. In terms of health, 79 of the 161 good-health-condition workers (49.1%) met satisfactory standards, whereas 23 of the 43 workers (53.5%) who perceived themselves to have uncertain or poor health condition reached this level. As for financial status, of the 117 older workers who were satisfied with their financial status, 57 performed satisfactorily. However, only 45 of the older workers who were dissatisfied financially were able to perform satisfactorily in the eyes of employers/supervisors.

### 4.2. Empirical Findings from Gologit Estimation

Data were first examined using the ordered logit (ologit) regression. However, unfortunately, ologit turned out to be inappropriate for our analysis when taking into consideration the proportionality assumption test. As outlined in the Brant test, presented in Table 3, the result from the ologit violates the proportionality assumption at a significant level of 0.05 (*P* > $\chi^{2}$ = 0.004), rejecting the null hypothesis. This implies that the ologit probably provides biased estimators. Thus, as a result, the gologit model was appropriately chosen as our analytical technique.

Then, we computed the data by utilizing the gologit model. Table 4 summarizes and presents the odd ratio from the gologit regression. It indicates that gender and age are the only factors that could not be a potential factor in determining older workers’ ability to satisfy employer expectations, both in indifferent (AP > 1) and higher levels (AP > 2) as compared to younger labor. Thus, gender and age factors are weak predictors of achieving satisfactory levels of productivity. However, other variables, namely, educational level, type of employment, occupational characteristics, health, and financial factors, do significantly satisfy productivity expectations at the *p*-value 0.05. 

In terms of educational level, boosting one’s educational level from below undergraduate to bachelor or graduate level will likely increase the odds of an older worker’s productivity rising above the dissatisfied level (AP > 1) by 2.91 times on average, holding other variables constant. This indicates that an older worker who has an undergraduate education or above tends to be more productive. However, this factor will not be significant at the highest level (AP > 2).

As for type of employment, at an indifferent level (AP > 1), the odd’s results indicate that, on average, older individuals who work full-time tend to be more productive than part-time workers (odd ratio = 4.78 > 1), holding other variables constant. However, focusing only on older workers who are at a satisfied level (AP > 2), it is observed that they differ from the indifferent level by being more productive when they work on a part-time basis (odd ratio = 0.34 < 1). This suggested part-time employment is expected to provide higher productivity than full-time basis. Flexibility in working might be its reason. A look at occupational characteristics indicates that when older workers perform general tasks, they do so at an indifferent level of productivity, reflected by an odd ratio < 1. However, when they work at tasks related to intensive communication and coordination and at technical tasks, they do so more productively, as measured by a satisfactory productivity level (odd ratio > 1). 

As for health, at an indifferent level (AP > 1), it is surprising that older workers who perceive their health to be only of a moderate level tend to be more significantly productive compared to those whose health is self-perceived as “good” (odd ratio < 1). This might reflect their motivation to maintain their health status by keeping active compared to those whose health is perceived as “good” already, thus resulting in a boost in their productivity. Finally, for financial status with odd ratio 2.25, we see that older workers who are satisfied with their financial status tend to be more productive than those concerned with their finances above the indifferent level (AP > 1). This lack of concern/worry about their finances/financial obligations might allow them to focus better on their work and thus be more productive. 

## 5. Discussion

As we realized that measuring productivity in labor can be varied due to differences in calculation method, there are some issues that can be observed the older population regarding productivity, in particular its characteristics profile and skills. 

It is noteworthy that our main conclusion revealed that gender had no apparent influence on productivity at the indifferent level and above (AP > 1 and AP > 2). This inference suggested that older workers, whether they are male or female, may likely produce a similar level of productivity. This reinforces the main conclusions of Göbel and Zwick [22], who showed that gender differences among service industry workers had only a minimal effect. More importantly, at an indifferent level and above (AP > 1), it was observed in the service sectors that the age of the worker is less significant to the work productivity. This is in line with key findings from Göbel and Zwick [22]. More importantly, we found positively preferable factors of older worker characteristics can better satisfy their employer/supervisor, supporting results from the previous studies including with a higher educational level [30], specific or technical knowledge-intensive [29], good health [27], and good financial factors [31]. 

At a high satisfied level, our key findings support Göbel and Zwick [22] who found that working on a full-time basis would be less productive in the service sector. This is particularly the case when those workers are older persons, and our findings addressed that working on a full-time basis would generate less productivity than on a part-time basis (odd ratio < 1). Flexibility preference might be their main reason for choosing part-time work, particularly when concerns about physical health or familial obligations impact their ability to work full time.

In addition to that, we, along with suggestions from Turek and Perek-Bialas [29], recommended an employer of older workers to take into account occupational characteristics. This factor would attract more older workers and help them to thrive and be productive in the workplace. One of the reasons older workers want to continue working or return to the labor market is that working after retirement can help them maintain better mental and physical health [27]. Thus, the older workers with this mindset will work with a positive attitude [27]. This is in line with the work productivity analysis that found that those working in intensive communication and coordination tend to reach a higher satisfactory level of employer expectation as compared to the those working at general tasks/work [29]. This implies that those older workers could contribute to boosting overall productivity when their duties require intense attitudes. Therefore, one approach for increasing productivity would be to allocate tasks to older workers that allow them to focus on communication or coordination, such as sales, customer service, or human resource management.

## 6. Conclusions and Recommendation

Utilizing evidence from Thailand, we have found that the productivity of older workers in the service sector is influenced by their educational level, type of employment, occupational characteristics, health, and financial status, rather than purely by gender and age factors. 

With emphasis on high productivity, the type of employment and occupational characteristics should be firmly taken into consideration, with special focus on allocating work related to communication and coordination to older workers which would enhance their productivity compared to the general task, as indicated by high odd ratio of the occurrent event (odd ratio 4.79). This is important for relevant stakeholders to take into consideration. More specifically, at high levels of productivity (AP > 2), key findings showed that part-time work increases productivity more than full-time employment (odd ratio = 0.34 < 1). This is probably affected by flexible working arrangements.

Productivity of older employees is an important factor influencing their employability since it reflects the value that they provide to the workplace. Therefore, effective strategies that enhance the productivity of the older workers must be developed. We would suggest that strategies should prioritize working basis, educational level, occupational characteristics, health, and finance, rather than gender and age. The key takeaways for promoting productivity in older workers are as follows:

- Increase gender equality in the workplace

This study has suggested that there is no significant gender effect on the productivity of the older workers. It implies that service tasks provided by males or females may have no difference. This challenges the existing world employment trend, in which most employers prefer to employ older females rather than older males, according to the employment statistics 1991–2019 reported by the world bank [48]. In this regard, promoting/advocating gender equality among older workers in a workplace should be taken into consideration by employers or respective agencies.

- Introduce vocationalization for pre-aging workers

It is acknowledged that occupational characteristics do predominantly affect productivity among the older workers. In this connection, there is a need for respective agencies to introduce vocational career training for pre-aging workers prior to their retirement. Increasing capacity with regard to required, relevant skills related to communication or coordination might be used as components in drafting their syllabi. This will help satisfy employer expectations when older workers try re-enter the labor market after retirement, particularly in the service sector.

- Increase motivation by reducing age-related obligations

This study provides evidence that motivations for working, such as concerns over health and financial status, could potentially impact the productivity among the older workers. Thus, there is a need to increase motivation by reducing age-related obligations. Ideally, this proposal should be initiated by employers, but entrusting this proposal to the government is foreseen to be less troublesome than entrusting it to employers. This is because employers are unlikely to implement any policies targeted at alleviating the burden of old age due to increasing operational costs, regardless of how destructive such old age burdens are to work productivity. As a result, the government would be a better, more reliable supporter of this initiative. In this regard, to increase motivation, effective tax incentives might be considered for those employers who do try to reduce old age obligations or increase the motivation of older workers, such as providing annual health checkups, soft loans, or a special savings rate for older workers.

## Figures and Tables

**Table 1:** Description of factors determining productivity.

No.	Variable	Description	
		Type of Variable	Details
1	Age	Numerical	Age is reported as a discrete number (year).
2	Gender	Dichotomous	Gender comprises male and female.
3	Education	Dichotomous	Education consisted of two levels: below undergraduate and undergraduate and above.
4	Type of employment	Dichotomous	Type of employment consists of two categories: full-time and part-time basis.
5	Occupational characteristics	Categorical	The variable highlighting the majority of the tasks/work that the employee has to deal with is divided into three categories i.e., General: work that does not require specific skills—security guard/maid, merchandiser, cashier. Communication and coordination-intensive: work that requires strong communication and coordination skills—customer services, sales representative, coordinator. Specific or technical knowledge-intensive: work that requires technical knowledge—consultant, inspector, manager.
6	Self-perceived health status	Dichotomous	Representing employees’ perception of their health status—good conditions or uncertainty/poor conditions.
7	Financial Status	Dichotomous	Representing employees’ perception of their financial status—satisfied (finances are sufficient to meet daily expenses) or otherwise/dissatisfied (finances are insufficient for meeting daily expenses).

Source: summarized by authors.

**Table 2:** Used data description.

Data	Observation		Productivity (*n*)					
			Dissatisfied (AP = 1)		Indifferent (AP = 2)		Satisfied (AP = 3)	
	*n*	%	*n*	%	*n*	%	*n*	%
Gender								
Male	76	37.3	28	36.8	10	13.2	38	50.0
Female	128	62.7	51	39.8	13	10.2	64	50.0
Age								
Lower than 60 years old	28	13.7	10	35.7	2	7.1	16	57.1
60–64 years old	115	56.4	44	38.3	10	8.7	61	53.0
65 years old and above	61	29.9	25	41.0	11	18.0	25	41.0
Education level								
Below undergraduate	148	72.5	60	40.5	18	12.2	70	47.3
Undergraduate and above	56	27.5	19	33.9	5	8.9	32	57.1
Type of employment								
Full Time	113	55.4	50	44.2	5	4.4	58	51.3
Part Time	91	44.6	29	31.9	18	19.8	44	48.4
Occupation characteristics								
General task	108	52.9	42	38.9	30	27.8	36	33.3
Communication and coordination-intensive	56	27.5	16	28.6	18	32.1	22	39.3
Specific or technical knowledge-intensive	40	19.6	10	25.0	13	32.5	17	42.5
Self-perceived health status								
Good condition	161	78.9	63	39.1	19	11.8	79	49.1
Uncertain or poor condition	43	21.1	10	23.3	4	9.3	23	53.5
Financial Status								
Satisfied	117	57.3	43	36.8	17	14.5	57	48.7
Dissatisfied	87	42.7	36	41.4	6	6.9	45	51.7

Source: calculated by authors.

**Table 3:** Brant test.

	$\chi^{2}$	*P* $>\;\chi^{2}$	df
All variables	22.43	0.004	8

Source: Estimated by authors.

**Table 4:** Gologit regression results.

Variable	*P* (AP)			
	*P* (AP > 1)		*P* (AP > 2)	
	Odd Ratio	*p*-Value	Odd Ratio	*p*-Value
Gender				
Female (based variable)				
Male	1.63	0.17	1.31	0.43
Age	1.02	0.73	0.93	0.11
Education level				
Below undergraduate (based variable)				
Undergraduate and above	2.91	0.02 *	1.25	0.54
Type of employment				
Part Time (based variable)				
Full Time	4.78	0.00 *	0.34	0.02*
Occupational characteristics				
General task (based variable)				
Communication and coordination-intensive	0.39	0.06	4.79	0.00 *
Specific or technical knowledges-intensive	0.33	0.03 *	2.69	0.05 *
Self-perceived health status				
Moderate (based variable)				
Good	0.25	0.01 *	1.23	0.61
Financial Status				
Dissatisfied (based variable)				
Satisfied	2.25	0.05 *	0.52	0.07
Constant	0.46	0.80	102.80	0.11
**Pseudo R2 = 0.1384** **Observation = 204**				

Remark: * Significant at level 0.05. Source: Authors calculated from Stata software in command gologit2, which is technically developed equivalent to gologit model [47].

## Data Availability

Not applicable.

## References

[1] Aiyar M.S., Ebeke M.C.H. (2016). The Impact of Workforce Aging on European Productivity.

[2] Dallmeyer S., Wicker P., Breuer C. (2017). How an aging society affects the economic costs of inactivity in Germany: Empirical evidence and projections. Eur. Rev. Aging Phys. Act..

[3] Fukuda S.-I., Okumura K. (2021). The aging society, savings rates, and regional flow of funds in Japan. J. Jpn. Int. Econ..

[4] Osatis C., Asavanirandorn C. (2022). An Exploring Human Resource Development in Small and Medium Enterprises in Response to Electric Vehicle Industry Development. World Electr. Veh. J..

[5] Costa G., Di Milia L. (2008). Aging and Shift Work: A Complex Problem to Face. Chronobiol. Int..

[6] Zacher H., Kooij D., Beier M.E. (2018). Active aging at work. Organ. Dyn..

[7] Alley D., Crimmins E., Shultz K.S., Adams G.A. (2007). The demography of aging and work. Aging and Work in the 21st Century.

[8] Vaupel J.W., Loichinger E. (2006). Redistributing Work in Aging Europe. Science.

[9] Suwanrada W. (2008). Poverty and Financial Security of the Elderly in Thailand. Ageing Int..

[10] Burtless G. (2013). The Impact of Population Aging and Delayed Retirement on Workforce Productivity (Working Paper 2013-11).

[11] Manyika J., Woetzel J., Dobbs R. (2015). Global Growth: Can Productivity Save the Day in an Aging World.

[12] Shephard R.J. (2000). Aging and productivity: Some physiological issues. Int. J. Ind. Ergon..

[13] Maestas N., Mullen K.J., Powell D. (2016). The effect of population aging on economic growth, the labor force and productivity. NBER Working Paper Series.

[14] Dostie B. (2011). Wages, productivity and aging. De Econ..

[15] Viviani C., Bravo G., Lavallière M., Arezes P., Martínez M., Dianat I., Bragança S., Castellucci H. (2021). Productivity in older versus younger workers: A systematic literature review. Work.

[16] Kenny D.T., Driscoll T., Ackermann B. (2018). Effects of Aging on Musical Performance in Professional Orchestral Musicians. Med. Probl. Perform. Artist..

[17] Siegel I.H. (1981). Productivity measurement at the firm Level. Productivity Analysis at the Organizational Level.

[18] Bitran G.R., Chang L. (1984). Productivity Measurement at the Firm Level. Interfaces.

[19] Tolentino A. New concepts of productivity and its improvement. Proceedings of the European Productivity Network Seminar.

[20] Maroto-Sánchez A. (2012). Productivity in the services sector: Conventional and current explanations. Serv. Ind. J..

[21] Cristea M., Noja G.G., Stefea P., Sala A.L. (2020). The Impact of Population Aging and Public Health Support on EU Labor Markets. Int. J. Environ. Res. Public Health.

[22] Göbel C., Zwick T. (2011). Age and Productivity: Sector Differences. De Econ..

[23] Ozimek A., DeAntonio D., Zandi M. (2018). Aging and the Productivity Puzzle.

[24] Park C.-Y., Shin K., Kikkawa A. (2021). Aging, automation, and productivity in Korea. J. Jpn. Int. Econ..

[25] Pfeifer C., Wagner J. (2014). Age and gender effects of workforce composition on productivity and profits: Evidence from a new type of data for German enterprises. Contemp. Econ..

[26] Said M., Galal R., Joekes S., Sami M. (2018). Gender Diversity, Productivity, and Wages in Egyptian Firms.

[27] Sewdas R., De Wind A., Van Der Zwaan L.G., Van Der Borg W.E., Steenbeek R., Van Der Beek A.J., Boot C.R. (2017). Why older workers work beyond the retirement age: A qualitative study. BMC Public Health.

[28] Skirbekk V. (2008). Age and productivity potential: A new approach based on ability levels and industry-wide task demand. Popul. Dev. Rev..

[29] Turek K., Perek-Bialas J. (2013). The role of employers opinions about skills and productivity of older workers: Example of Poland. Empl. Relat..

[30] Van Dalen H.P., Henkens K., Schippers J. (2010). Productivity of Older Workers: Perceptions of Employers and Employees. Popul. Dev. Rev..

[31] Kaur S., Mullainathan S., Oh S., Schilbach F. (2021). Do Financial Concerns Make Workers Less Productive? Working Paper 26110.

[32] Börsch-Supan A., Hunkler C., Weiss M. (2021). Big data at work: Age and labor productivity in the service sector. J. Econ. Ageing.

[33] Abbey E., Adu-Danso E. (2022). Gender diversity and productivity in manufacturing firms: Evidence from six Sub-Saharan African (SSA) countries. J. Manag. Organ..

[34] Amutabi C. (2019). Factors Influencing Labor Productivity in The Kenyan Services Sector.

[35] Amutabi C., Wambugu A. (2020). Determinants of labor productivity among SMEs and large-sized private service firms in Kenya. Afr. Dev. Rev..

[36] Heshmati A., Rashidghalam M., Heshmati A. (2018). Labour productivity in Kenyan manufacturing and service industries. Determinants of Economic Growth in Africa.

[37] Børing P., Grøgaard J.B. (2021). Do older employees have a lower individual productivity potential than younger employees?. J. Popul. Ageing.

[38] Breinek P. (2018). Problems of older workers on the labour market. Acta Univ. Agric. Silvic. Mendel. Brun..

[39] Bryson A., Forth J., Gray H., Stokes L. (2020). Does Employing Older Workers Affect Workplace Performance?. Ind. Relat. A J. Econ. Soc..

[40] Asavanirandorn C., Osatis C., Pechdin W. (2020). Non-Standard Employment for Older Persons in the Thai Private Sector: Flexibility Productivity and Protection.

[41] Brant R. (1990). Assessing Proportionality in the Proportional Odds Model for Ordinal Logistic Regression. Biometrics.

[42] Dolgun A.B., Saracbasi O. (2014). Assessing proportionality assumption in the adjacent category logistic regression model. Stat. Interface.

[43] Fu V.K. (1998). sg88: Estimating generalized ordered logit models. Stata Technical Bulletin Reprints.

[44] Anh N., Nguyen N.M.T., Anh N.T.T., Nguyen P.M.T. (2019). Job satisfaction in developing countries: An evidence from a matched employer–employee survey in Vietnam. J. Econ. Stud..

[45] Bartoll X., Ramos R. (2020). Worked hours, job satisfaction and self-perceived health. J. Econ. Stud..

[46] Abrudan I.-N., Pop C.-M., Lazăr P.-S. (2020). Using a General Ordered Logit Model to Explain the Influence of Hotel Facilities, General and Sustainability-Related, on Customer Ratings. Sustainability.

[47] Williams R. (2006). Generalized Ordered Logit/Partial Proportional Odds Models for Ordinal Dependent Variables. Stata J. Promot. Commun. Stat. Stata.

[48] The World Bank Employment in Services, Female (% of Female Employment) (Modeled ILO Estimate). https://data.worldbank.org/indicator/SL.SRV.EMPL.FE.ZS.

